# Tanreqing Injection Attenuates Macrophage Activation and the Inflammatory Response *via* the lncRNA-SNHG1/HMGB1 Axis in Lipopolysaccharide-Induced Acute Lung Injury

**DOI:** 10.3389/fimmu.2022.820718

**Published:** 2022-04-25

**Authors:** Chunling Hu, Junlu Li, Yingshuai Tan, Yang Liu, Chen Bai, Jing Gao, Shilong Zhao, Mengying Yao, Xiaoxiao Lu, Lingxiao Qiu, Lihua Xing

**Affiliations:** ^1^ Department of Respiratory Intensive Care Unit, The First Affiliated Hospital of Zhengzhou University, Zhengzhou, China; ^2^ School of Traditional Chinese Medicine, Beijing University of Chinese Medicine, Beijing, China; ^3^ Department of Respiratory Medicine, The First Affiliated Hospital of Zhengzhou University, Zhengzhou, China

**Keywords:** acute lung injury/acute respiratory distress syndrome (ALI/ARDS), macrophage polarization, tanreqing injection, NF-κB signaling pathway, long noncoding RNA (lncRNA), high mobility group protein 1 (HMGB1)

## Abstract

The etiology of acute lung injury (ALI) is not clear, and the treatment of ALI presents a great challenge. This study aimed to investigate the pathogenesis and potential therapeutic targets of ALI and to define the target gene of Tanreqing (TRQ), which is a traditional Chinese medicine formula composed of five medicines, scutellaria baicalensis, bear bile powder, goat horn powder, honeysuckle and forsythia. Macrophage activation plays a critical role in many pathophysiological processes, such as inflammation. Although the regulation of macrophage activation has been extensively investigated, there is little knowledge of the role of long noncoding RNAs (lncRNAs) in this process. In this study, we found that lncRNA-SNHG1 expression is distinctly regulated in differently activated macrophages in that it is upregulated in LPS. LncRNA-SNHG1 knockdown attenuates LPS-induced M1 macrophage activation. The SNHG1 promoter was bound by NF-κB subunit p65, indicative of SNHG1 being a direct transcriptional target of LPS-induced NF-κB activation. SNHG1 acts as a proinflammatory driver that leads to the production of inflammatory cytokines and the activation of macrophages and cytokine storms by physically interacting with high-mobility group box 1 (HMGB1) in ALI. TRQ inhibited NF-κB signaling activation and binding of NF-κB to the SNHG1 promoter. In conclusion, this study defined TRQ target genes, which can be further elucidated as mechanism(s) of TRQ action, and provides insight into the molecular pathogenesis of ALI. The lncRNA-SNHG1/HMGB1 axis is an ideal therapeutic for ALI treatment.

## Introduction

Acute lung injury (ALI) and acute respiratory distress syndrome (ARDS) are acute diffuse inflammatory lung injuries characterized by intractable hypoxemia and rapid clinical progress and caused by various intrapulmonary and extrapulmonary pathogenic factors, such as sepsis, pancreatitis, multiple traumas, pneumonia, aspiration of gastric contents, and pulmonary contusion (1). Patients with ALI and ARDS can quickly develop multiple organ failure (MODF), with a fatality rate as high as 30% to 50% (2). At present, the main treatment for ARDS mainly focuses on respiratory support and etiological treatment. Although respiratory support methods such as extracorporeal membrane oxygenation (ECMO) have developed rapidly in recent years, the short-term and long-term mortality of ARDS patients has not been significantly improved (3). Therefore, ideal drugs and therapeutic means to effectively curb the inflammatory response of ARDS have become urgent treatment problems to be solved.

The characteristic advantage of traditional Chinese medicine treatment (TCM) treatment is to mobilize the patient’s own adaptation, reconciliation and disease resistance and treat according to syndrome differentiation (4). It can fight inflammation through multiple targets, multiple links, and multiple pathways and protect against lung injury. But, TCM has always paid attention to its medicinal properties and efficacy, but the research on its medicinal ingredients, mechanism of action and pharmacokinetics is very weak, so the understanding of the toxic and side effects of TCM is insufficient (5, 6). In recent years, clinical research and animal experiments of traditional Chinese medicine in the treatment of ALI/ARDS have intensified, and certain results have been achieved. Tanreqing injection (TRQ) is a traditional Chinese medicine formula composed of five medicines: Scutellariae Radix, Fel selenarcti, Cornu naemorhedi, Lonicerae japonicae Flos, and Forsythiae fructus. It has antibacterial, anti-inflammatory, expectorant, anti-convulsant and antipyretic effects. At present, research on the mechanism of TRQ is still limited. Studies have found that in RAW264.7 macrophages treated with LPS, Scutellaria baicalensis in Tanreqing prescription inhibits the secretion of the proinflammatory cytokines Cox2 and iNOS through NF-κB (4), thereby inhibiting the inflammatory reaction. In the ALI rat model, TRQ had a significant regulatory effect on the downstream IL-17 and MUC5AC proteins and significantly reduced the levels of TNF-α, IL-6, IL-17A and other proinflammatory cytokines (7). The above studies suggest that TRQ may have an exciting anti-inflammatory effect on the inflammatory response in ALI/ARDS, but the mechanism of action needs to be further elucidated.

Macrophages constitute an essential element of the innate and adaptive immune systems, and as the control switch of the immune system, they maintain the balance between proinflammatory and anti-inflammatory responses. Macrophages show different functional phenotypes in different microenvironments, which can be polarized into classically activated M1 macrophages and alternatively activated M2 macrophages. M1 macrophages produce high amounts of proinflammatory cytokines and are critical to the eradication of bacterial, viral and fungal infections (8–10). The latest research has found that transcriptional and epigenetic levels are profoundly associated with macrophage activation and then promote numerous pathologies (8, 11). Long noncoding RNAs (lncRNAs) are a large class of nonprotein-coding transcripts that are greater than 200 bases in length and have confirmed the relevance of lncRNAs to respiratory disease. LncRNA small nucleolar RNA host gene 1 (SNHG1) is a newly defined lncRNA located on chromosome 11q12.3. The expression of SNHG1 is strongly regulated in lung adenocarcinoma (12), hepatocellular carcinoma (13), bladder cancer (14), glioma (15), and other physiological processes. In particular, the overexpression of SNHG1 has been reported to compete endogenous RNA for miR-7 to regulate NLRP3 expression, leading to the activation of the NLRP3 inflammasome and promoting neuroinflammation in Parkinson’s disease (16). The molecular mechanisms by which SNHG1 exerts its biological functions are diverse and complex and guide the regulation of protein–DNA interactions (17) or miRNA sponges (18). While there is plenty of evidence showing that noncoding RNAs participate in macrophage activation by targeting various mediators, the mechanisms underlying these effects are still unknown.

In this study, we defined SNHG1 as an important transcription factor that promotes the expression of proinflammatory cytokines in macrophages and in mice. SNHG1 is expressed in macrophages with LPS but downregulated in cells stimulated with TRQ. SNHG1 is significantly associated with inflammatory gene expression and exerts its biological function through the proinflammatory gene NF-kB-SNHG1-HMGB1 axis in ALI cells. Our data suggested that SNHG1 is a potentially robust biomarker and that TRQ has therapeutic efficacy against inflammation.

## Materials and Methods

### Cell Lines and Reagents

RAW264.7, THP1 and HEK293T cells were obtained from the American Type Culture Collection (ATCC, Manassas, Virginia, USA). Mouse BMDMs were derived from bone marrow cells of C57BL/6 mice as previously described (19). Briefly, the mice were killed by cervical dislocation and then soaked in 75% ethanol. Then, femurs and tibias were harvested, and bone marrow cells from all bones were flushed out. Then, after centrifugation for 5 min at 310 × g and erythrocyte lysis, the remaining cells were seeded in plates and cultured in DMEM containing 10% FBS and 50 ng/ml recombinant mouse macrophage colony-stimulating factor (M-CSF) for 7 days to form proliferative nonactivated cells (also named M0 macrophages). To establish human macrophages, THP-1 monocytes differentiated into macrophages by 36 h incubation with 150 nM phorbol 12-myristate 13-acetate (PMA, Sigma, P8139) in RPMI medium containing 10% FBS. Once differentiated (M0 macrophages), they were incubated with human recombinant IFN-γ (50 ng/ml, PeproTech, #300-02-20UG) in order to obtain classical macrophage activation (M1) and with human IL-4 (40 ng/ml, PeproTech, #200-04) for M2 polarized macrophages. RAW264.7 and HEK293T cells were cultured in DMEM containing 10% FBS and 1% penicillin-streptomycin solution (100×). Ultra-pure LPS from Escherichia coli O111:B4. Tanreqing (TRQ) injection was purchased from Shanghai Kai Bao Pharmaceutical Co., Ltd. (Shanghai, China). Mouse IL-4 (PeproTech, #214-14) was obtained from PeproTech.

### Mice

Male C57BL/6 mice free of murine-specific pathogens were purchased from Beijing Vital River Laboratory Animal Technology Co., Ltd. The mice were raised in an SPF grade room with a 12-h dark/light cycle and granted free access to water and food at the Henan Experimental Animal Center. All animal experiments complied with the ethical guidelines of the Administration of Laboratory Animals of the Experimental Animal Center of Zhengzhou University, and the study protocol was approved by the Ethics Committee of the First Affiliated Hospital of Zhengzhou University.

### Acute Lung Injury Model

All mice were allowed a minimum facility acclimatization period of 7 days before being subjected to experiments. Healthy male C57BL/6 mice were randomly divided into six groups (n=5): the control group, LPS group, TRQ group (8 ml/kg), LPS+ TRQ (low concentration, 4 ml/kg, LTL) group, LPS+ TRQ (medium concentration, 6 ml/kg, LTM) group, and LPS+ TRQ (high concentration, 8 ml/kg, LTH) group. The mice were anesthetized through intraperitoneal administration of sodium pentobarbital (50 mg/kg). Then, the mice were intratracheally instilled with 7 mg/kg LPS in 50 μL of phosphate-buffered saline (PBS) or sterile PBS alone (control group), after which TRQ was administered intraperitoneally (i.p.). All mice were sacrificed following the collection of whole blood, bronchoalveolar lavage fluid (BALF), and lung tissues 24 h after LPS challenge.

### Reverse Transcription PCR and Quantitative Real-Time PCR

First, RNA samples from mouse tissue specimens and cell lines were used in this study and extracted with TRIzol reagent (Takara, #9108). Second, cDNA was synthesized using the PrimeScript™ RT Reagent Kit with gDNA Eraser (Takara, RR047A). Finally, relative RNA levels were determined by quantitative real-time PCR (qRT-PCR) using the QuantStudio™ 5 System. qRT-PCR was performed using TB Green^®^ Premix Ex Taq™ II (Takara, RR820A) according to the manufacturer’s instructions. The primer sequences are shown in **Table 1**.

**Table 1:** Primer sequences for human genes.

Gene	Forward primer (5’→3’)	Reverse primer (5’→3’)
GADPH	5’-AAGGGCTCATGACCACAGTC-3’	5’-ATCACGCCACAGCTTTCCA-3’
SNHG1	5’-AGCAGACACAGATTAAGACA-3’	5’-GGCAGGTAGATTCCAGATAA-3’
Gene	Forward primer (5’→3’)	Reverse primer (5’→3’)
GADPH	5’-GGTGAAGGTCGGTGTGAACG-3’	CTCGCTCCTGGAAGATGGTG-3’
SNHG1	5’-TCCCAGGATGAGTGCAGGTT-3’	TCCTGGTACGGCTCCTTTGT-3’
TNF-α	5’-CATCTTCTCAAAATTCGAGTGAC-3’	TGGGAGTAGACACAAGGTACAA
IL-6	5’-CTGCAAGAGACTTCCATCCAG-3’	AGTGGTATAGACAGGTCTGTTGG-3’
IL-10	5’-GCCCAGAAATCAAGGAGCATT-3’	CGCATCCTGAGGGTCTTCA-3’
F4/80	5’-TGACTCACCTTGTGGTC-CTAA-3’	5’-CTTCCCAGAATCCAGTCTTTCC-3’
MCP-1	5’-GTTAACGCCCCACTCACCTG-3’	5’-GGGCCGGGGTATGTAACTCA-3’
iNOS	5’-CAGGGCCACCTCTACATTTG-3’	5’-TGCCCCATAGGAAAAGACTG-3’
Arg-1	5’-ACATTGGCTTGCGAGACGTA-3’	5’-ATCACCTTGCCAATCCCCAG-3’
IL-1β	5’-GTCGCTCAGGGTCACAAGAA-3’	5’-GTG-CTGCCTAATGTCCCCTT-3’

### RNA Interference and Generation of Lentiviral Particles

Full-length SNHG1 was cloned into overexpression lentivirus (GeneChem, Shanghai, China), while the mock vector with no SNHG1 sequence served as a control. To generate adenovirus expressing shRNA against SNHG1 (Ad-sh-SNHG1), 3 siRNAs for mouse SNHG1(GenePharma, Shanghai, China) were designed, and the shRNA with the optimal knockdown efficiency was chosen to create shRNA and then recombined into adenoviral vectors. The target sequence is as follows: CCATAAGAGATCACTTTAA. The negative control adenovirus was designed to express nontargeting “universal control” shRNA (Ad-shNC). To generate adenovirus expressing shRNA against HMGB1 (Ad-sh-HMGB1), 2 siRNAs (sh-HMGB1-551 and sh-HMGB1-773) for mouse HMGB1 (GenePharma, Shanghai, China) were designed, and the shRNA with the optimal knockdown efficiency was chosen to create shRNA and then recombined into adenoviral vectors. The target sequence is as follows: CAAGGCTCGTTATGAAAGAGA (LV3-HMGB1-551) and GCAGCCCTATGAGAAGAAAGC (LV3-HMGB1-551). The negative control adenovirus was designed to express nontargeting “universal control” shRNA (Ad-shNC). Amplification and purification of recombinant adenovirus was performed according to the manufacturer’s instructions.

### ELISA

The concentrations of TNF-α, IL-6, and HMGB1 in the cell supernatant, plasma and BALF from animals were calculated by using ELISA kits according to the manufacturer’s instructions. ELISA kits were TNF-α, (CSB-E04741M-96, CUSABIO, Wuhan, China), IL-6 (CSB-E04639m, CUSABIO, Wuhan, China), and HMGB1 (CSB-E08225m, CUSABIO, Wuhan, China).

### Western Blot

Total protein was extracted from cells or tissues by RIPA buffer at the indicated times and homogenized in ice-cold suspension buffer supplemented with a proteinase inhibitor. Protein concentrations were determined using the BCA Protein Assay Kit (Solarbio, Beijing, China). Equal amounts of protein were fractionated by on SDS-PAGE gels and then transferred to PVDF membranes (Millipore, Massachusetts, USA), followed by immunoblotting with the following primary antibodies: Anti-NF-kB p65 antibody (Rabbit monoclonal antibody, diluted at 1:5000, ab32536, Abcam); Anti-NF-kB p65 (phospho S536) (Rabbit monoclonal antibody, diluted at 1:1000, ab76302, Abcam); Anti-p38 alpha/MAPK14 antibody (Rabbit monoclonal antibody, diluted at 1:3000, ab170099, Abcam); Phospho-p38 MAPK (Thr180/Tyr182) (Rabbit monoclonal antibody, diluted at 1:1000, #4511, CST); Anti-HMGB1(Rabbit monoclonal antibody, diluted at 1:10000, ab79823, Abcam); GAPDH (Rabbit monoclonal antibody, diluted at 1:5000, Proteintech, Wuhan, China); and Beta Actin antibody (Rabbit monoclonal antibody, diluted at 1:5000, Abways Technology, Shanghai, China). After blocking with 5% BSA in TBST, PVDF membranes were incubated with primary antibodies at 4°C overnight and then hybridized with a secondary antibody at 37°C for 1 h. The intensity of the bands was analyzed with a chemiluminescence kit (Millipore, Massachusetts, USA).

### Chromatin Immunoprecipitation (ChIP) Assay

ChIP assays were performed as previously described (20). Briefly, ten million RAW264.7 cells were washed twice in ice-cold PBS buffer, cross-linked with 1% formaldehyde for 10 min at room temperature and then quenched by the addition of glycine (125 mmol/L final concentration). Then, chromatin was sonicated to obtain soluble sheared chromatin (average DNA length of 200–500 bp). Twenty microliters of chromatin were saved at –20°C for input DNA, and 100 µl of chromatin was used for immunoprecipitation by anti-NF-kB-p65 antibodies (CST, #8242). Genomic DNA in the immunocomplexes was purified using Qiagen miniprep columns. The primer sequences used to specifically amplify the mouse SNHG1 promoter spanning the putative NF-κB binding site were Forward primer: CTTTGGGCGGATTCCTCTAC, Reverse primer: GGGATTTGGAGGTGGGACT.

### Nuclear and Cytoplasmic RNA Extraction

The nuclear and cytoplasmic fractions of RNA were extracted with a PARIS™ kit (Invitrogen, Thermo Fisher Scientific, Waltham, USA). The cells were placed at the bottom of a 25 T culture flask and cultivated to an appropriate number (1× 10^7^/well), and the following experimental steps were performed according to the manufacturer’s instructions.

### Fluorescence *In Situ* Hybridization (FISH)

Put the sterilized climbing piece into a 6-well plate, and then drop 1ml of cell suspension (about 5x105 cells) on the climbing piece. After 1 hour, gently add 1ml of medium and place it in a 37°C 5% CO2 incubator overnight. The cells will grow well on the climbing piece. The next day, they will be treated with LPS. After 24 hours, the climbed slide was washed twice with PBS solution, and 1ml of 4% paraformaldehyde solution was added to each well of the 6-well plate, and then 500ul PBS was added for subsequent experiments. FISH assay was executed to observe the location of SNHG1 in RAW264.7 cells and LPS induced-RAW264.7 cells. Briefly, after prehybridization at 37°C for 30 min, cell climbing piece were hybridized with 2.5 μL 20 μM specific Cy3-labelled SNHG1 probes (Servicebio, Wu-han, China) at 37°C overnight, and dyed with DAPI. The probe sequences were TTCCTATCCTTCACACGCAGCTCATTCTTTTCCTC. Slides were photographed with confocal laser scanning microscopy (Zeiss, Jena, Germany).

### CCK-8 and Apoptosis

Cell Counting Kit-8 (Dojindo Laboratories, Kumamoto, Japan) assays were used to detect the proliferation of RAW264.7 cells according to the manufacturer’s instructions. The cells were placed at the bottom of a 96-well plate and incubated overnight. LPS and TRQ were added over a time gradient. Finally, CCK-8 reagent was added, and a microplate reader was used to detect cell viability. RAW264.7 cell apoptosis was detected by an Annexin V-FITC/propidium iodide (PI) apoptosis detection kit (KeyGEN, Jiangsu, China). The cells were harvested and double stained with FITC and PI after stimulation with different concentrations of TRQ, followed by analysis on a flow cytometer (Becon Dickinson FACSCalibur, NY, USA). Reactive oxygen species (ROS) assay kit (Beyotime, Shanghai, China) were used to detect ROS of RAW264.7 cells according to the manufacturer’s instructions. Place the cells at the bottom of the 96-well plate, followed overnight and adding in the LPS and TRQ. Dilute DCFH-DA 1:1000 with serum-free medium to make the final concentration 10umol/L. Remove the cell culture medium and add an appropriate volume of diluted DCFH-DA. Incubate in in a 37°C 5% CO2 incubator for 20 minutes. Wash the cells 3 times with serum-free cell culture medium. 96-well plate were photographed with Inverted microscope (OLYMPUS, Japan).

### Bacteria Killing Assay

A bacterial killing assay was performed as described previously (21). Briefly, 1 x 10^8^ cfu/ml Escherichia coli (BL21DE3pLysS) was added to macrophages in 6-well plates and incubated at 37°C for 1 h. Then, the supernatant was collected and centrifuged at 5000 rpm for 5 min. The supernatant was subsequently diluted 3 times, and the cells were plated on Luria broth-agar plates. The plates were incubated overnight at 37°C, and bacterial colonies were counted. Data are presented as the colony-forming unit (cfu)/ml.

### RNA Sequencing

We used RAW264.7 cells transfected with sh-SNHG1, and total RNA samples were collected using TRIzol reagent. RNA-seq was performed by Inheregene Co., Ltd. (Hangzhou, China).

### Chromatin Isolation by RNA Purification (ChIRP) and RNA Immunoprecipitation (RIP) Assay

Cells were seeded at the bottom of a 25 T culture flask and cultivated to an appropriate number (1× 10^7^/well). Then, the cells were harvested, and 240 mJ to 960 mJ was used for cell cross-linking, followed by cell lysis and ultrasonic chromosome fragmentation. A biotin-labeled DNA (20 bp) probe complementary to the target RAN sequence with one probe per 100 nt or so was synthesized. Finally, the RNA and protein were eluted. RNA was reverse transcribed into cDNA, and then the gene product was detected. Proteins were silver stained and detected by mass spectrometry. According to the manufacturer^’^s instructions of Magna RIP RNA-Binding Protein Immunoprecipitation Kit (Millipore), RIP experiments were conducted with HMGB1 antibody (Abcam, #ab228624) and immunoglobulin G antibody (IgG; #ab109489). Co-precipitated SNHG1 was applied to qRT-PCR.

### Data Mining and Analysis

One independent cohort of primary acute lung injury GEO dataset GSE2411 (22) was downloaded from the GEO database and analyzed in RStudio.

### Lung Wet/Dry(W/D) Weight Ratio Analysis

The wet/dry ratio is an indicator of pulmonary edema by calculating extravascular lung water. The right upper lung lobes were harvested and weighed as soon as possible to obtain the “wet weight”. Subsequently, tissues were dried in an oven at 65°C for 48 h and weighed again to calculate the “dry weight”. Lung wet/dry ratio = wet weight divided by dry weight.

### BALF Analysis

The right lower lung lobes and bronchoalveolar lavage fluid (BALF) were obtained by cannulating the trachea and then injecting and retrieving sterile 500 µl PBS two times. BALF was centrifuged at 1500 rpm at 4°C for 10 min to determine the concentrations of HMGB1, IL-6, and TNF-α.

### Hematoxylin and Eosin (HE), TUNEL Staining and Immunohistochemistry (IHC) Analysis

For histological examination, the left upper lung lobes of each mouse were fixed in 10% formalin and embedded in paraffin wax. Paraffin sections 5 µm thick were stained for HE and were reviewed by two skilled pathologists. To quantify lung injury and the inflammatory response, paraffin sections of each mouse were evaluated in 3 random fields (x200 magnification) using an inverted microscope (TE2000-U; Nikon Corporation, Tokyo, Japan). Then, alveolar edema, pulmonary hemorrhage, atelectasis and inflammatory cell infiltration were adopted in a semiquantitative histology score method, with each being scored on a 0–4 scale. The total score was calculated by adding the scores of all four histological indexes. Apoptotic cells in each paraffin section were detected by a TUNEL kit (Roche, Indianapolis, USA) according to the manufacturer’s instructions. For IHC, paraffin sections were incubated with primary antibodies against F4/80 (Cell Signaling Technology, USA) at 37°C for 60 min, secondary antibodies at 37°C for 15 min and horseradish enzyme-labeled streptavidin solution for 10 min, followed by staining with DAB and hematoxylin. Five 400x fields were randomly selected from each section, and each field was scored by staining intensity and percentage of positive cells. The product of staining intensity and percentage of positive cells was 0 score as negative (-), 1-4 score as weak positive (+), and 5-8 score as moderate positive (++).

### Lung Ultrasound of Mice

For lung injury examination, lung ultrasound was performed using a high-resolution Vevo2100 Ultrasound System (Visualsonics Inc., Toronto, Canada) with an ultrahigh-frequency (40 MHz) transducer probe to obtain a maximum resolution of 30 µm and an imaging depth of 10.0 mm. The hair of the anterior chest was removed from the mice by depilatory cream after 24 h and 48 h of exposure to LPS. Lung ultrasound videos were recorded and analyzed by two expert technicians (Shanshan Zhang and Xiaoxiao Zhang).

### Statistical Analysis

Data are shown as the mean ± standard deviation (SD) and were analyzed with IBM SPSS Statistics 21 (Chicago, IL, United States). Graphs were generated by GraphPad Prism 8.0 (GraphPad Software Inc., CA, USA). Student’s t-test was performed to analyze the differences between two groups and one-way ANOVA was performed to analyze the differences in more than two groups. All statistical tests were 2 sided, and *P* < 0.05 was considered statistically significant.

## Results

### Distinct Expression of lncRNA-SNHG1 in Differentially Activated Macrophages

**Figure 1:**
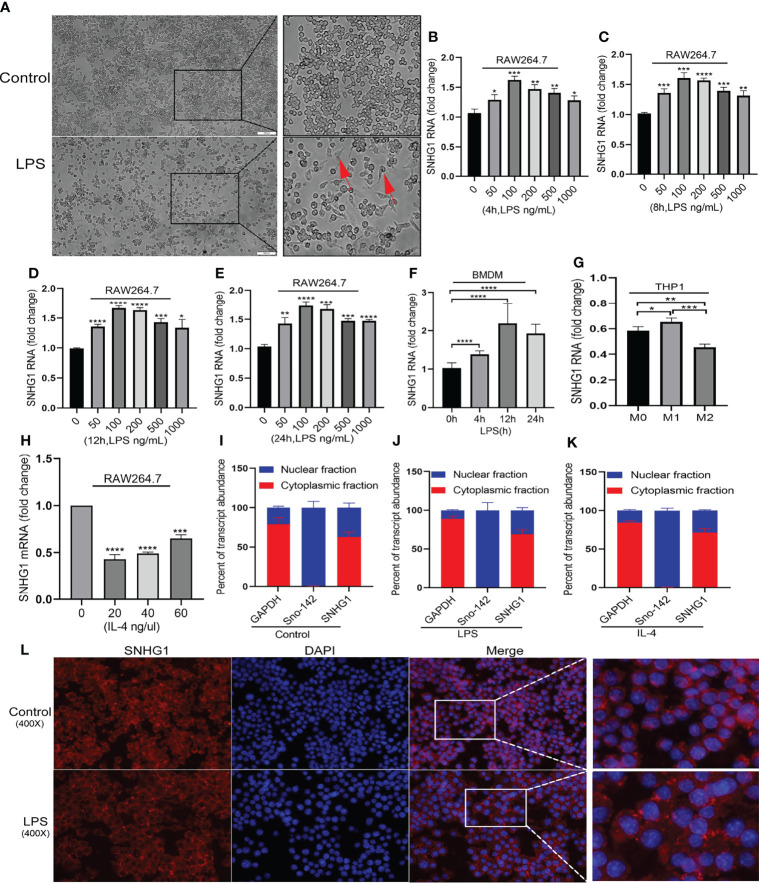
Distinctly expressed lncRNA-SNHG1 in differentially activated macrophages. **(A)** The morphology of RAW264.7 cells was significantly changed after LPS stimulation (magnification, ×100, scale bar, 100 μm). **(B–E)** The expression of SNHG1 in RAW264.7 cells treated with an LPS concentration gradient for 4 h, 8 h, 12 h, and 24 h. **(F)** Mouse BMDMs were treated with 100 ng/ml LPS for the indicated duration of time. Total RNA was isolated, and the levels of SNHG1 were determined by qRT-PCR. **(G)** Human THP-1-derived macrophages were treated with PMA, human IFN-r and IL-4 for the indicated times. Levels of SNHG1 were determined. **(H)** RAW264.7 cells were treated with a concentration gradient of mouse IL-4 for 24 h. Levels of SNHG1 were determined. **(I–K)** qRT-PCR was used to measure the level of SNHG1 in the nucleus and cytoplasm of RAW264.7 cells treated with PBS, LPS and IL-4. **(L)** FISH was performed to observe the cellular location of expression of SNHG1 (red) in RAW264.7 cells and in LPS induced-RAW264.7 cells (magnification, ×400, scale bar, 20μm and magnification, ×1000, scale bar, 10μm). The data between two groups were compared using unpaired t-tests. Data are indicated as the mean ± SD, *P<0.05, **P<0.01, ***P<0.001, ****P<0.0001.

To clarify the role of SNHG1 in macrophage activation, we initially determined the regulation of its expression by LPS and IL-4, which are classically used to induce the two polarized states of macrophages (23). After LPS stimulation, the morphology of the macrophages showed significant changes (**Figure 1A**). SNHG1 expression started to increase as early as 4 h after LPS treatment in RAW264.7 macrophages, and this elevation was persistent for at least 24 h (**Figures 1B**–**E**). The above results showed that 100 ng/mL is the optimal LPS concentration. SNHG1 was also induced in bone marrow-derived macrophages (BMDMs) at a later time point after LPS stimulation (**Figure 1F**), and SNHG1 was elevated after human monocytic line THP-1-derived macrophages were stimulated by PMA and IFN-r and suppressed by IL-4 (**Figure 1G**). In contrast to the LPS-treated cells, SNHG1 expression was inhibited by IL-4 in the RAW264.7 cells (**Figure 1H**). Taken together, the results showed that the expression of SNHG1 changed after stimulation with LPS and IL-4, suggesting that SNHG1 may participate in the differential activation of macrophages. To shed light on the mechanism underlying SNHG1 in macrophages, we determined the intracellular localization of SNHG1 by performing nuclear and cytoplasmic fractionation. According to previous research (17), GAPDH and the small nucleolar RNA Sno-142 are almost exclusively present in the cytoplasm and nuclei of macrophages. Thus, we determined the purity of the two cellular fractions (**Figures 1I**–**K**). We found that SNHG1 was virtually localized in the macrophage cytoplasm, and this predominant cytoplasmic localization remained unchanged after LPS or IL-4 treatment (**Figures 1I**–**K**). FISH assay also found that SNHG1 (red) was mainly collocated in cytoplasm in RAW264.7 cells and also collocated in cytoplasm in LPS induced-RAW264.7 cells (**Figure 1L**).

### Upstream Mediators of SNHG1 Expression in Macrophages

**Figure 2:**
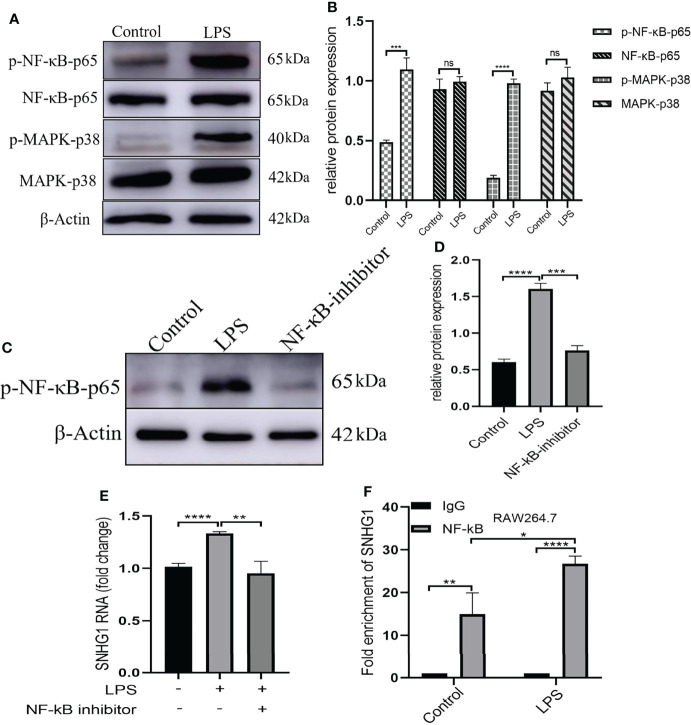
Upstream mediators of SNHG1 expression in macrophages. **(A)** Relative protein levels of p-NF-κB-p65, NF-κB-p65, p-MAPK-p38 and MAPK-p38 were detected in RAW264.7 cells treated with LPS by Western blot analysis. **(B)** The gray value of protein expression in the control and LPS groups. **(C)** Expression of p-NF-κB-p65 in RAW264.7 cells treated with an NF-κB inhibitor (BAY 11-7082) detected by Western blot analysis. **(D)** The gray value of protein expression in the control, LPS and NF-κB inhibitor groups. **(E)** The expression of SNHG1 in the PBS, LPS and LPS +NF-κB inhibitor groups. **(F)** Levels of p65 binding to the SNHG1promoter were determined by CHIP assay and the production of enrichment was determined by qRT-PCR. Data are indicated as the mean ± SD, ns *P*≥0.05, *P<0.05, **P<0.01, ***P<0.001, ****P<0.0001.

LPS acts through distinct pathways leading to macrophage activation. As shown in **Figures 2A, B**, we found that the expression of NF-κB-p65 and MARK-p38 were not significantly changed and only the phosphorylation of those two were significantly increased after LPS stimulation. Then, we selected pharmacological inhibitors of NF-κB (Bay-11-7082) to assess the functional consequences of inhibiting this pathway on SNHG1 expression. The dose of Bay-11-7082 was 5μM and was performed as previously described (24). The phosphorylation of NF-κB-p65 was significantly decreased after Bay-11-7082 stimulation (**Figures 2C, D**). Treatment with Bay-11-7082 completely abrogated LPS-induced SNHG1 upregulation in macrophages (**Figure 2E**). To further characterize the regulation of SNHG1 expression by NF-κB, we performed a ChIP assay and found that NF-κB subunit p65 bound to the SNHG1 promoter in LPS-treated macrophages and the bound was increased in LPS-induced RAW264.7 cells (**Figure 2F**), indicative of SNHG1 being a direct transcriptional target of LPS.

### SNHG1 Regulates Macrophage Polarization

**Figure 3:**
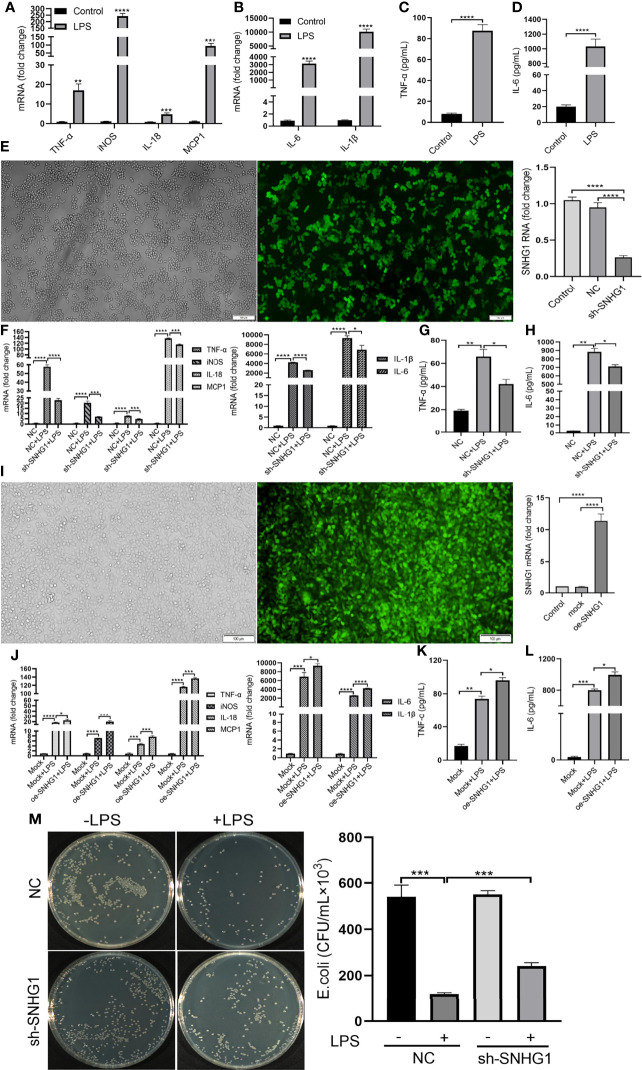
SNHG1 regulates macrophage polarization. **(A, B)** Expression of proinflammatory cytokines (TNF-α, iNOS, IL-18, MCP-1, IL-6 and IL-1β) in RAW264.7 cells treated with LPS, as determined by qRT-PCR. **(C, D)** Expression of proinflammatory cytokines (TNF-α, IL-6) in RAW264.7 cells treated with LPS, as determined by ELISA. **(E)** The efficiency of SNHG1 in RAW264.7 cells transfected with lentivirus sh-SNHG1 was determined by qRT-PCR. **(F)** Expression of proinflammatory cytokines (TNF-α, iNOS, IL-18, MCP-1, IL-6 and IL-1β) in RAW264.7 cells transfected with lentivirus sh-SNHG1 treated with LPS, as determined by qRT-PCR. **(G, H)** Expression of proinflammatory cytokines (TNF-α, IL-6) in RAW264.7 cells transfected with lentivirus sh-SNHG1 treated with LPS, as determined by ELISA. **(I)** The efficiency of SNHG1 in RAW264.7 cells transfected with lentivirus oe-SNHG1 was determined by qRT-PCR. **(J)** Expression of proinflammatory cytokines (TNF-α, iNOS, IL-18, MCP-1, IL-6 and IL-1β) in RAW264.7 cells transfected with lentivirus oe-SNHG1 treated with LPS, as determined by qRT-PCR. **(K, L)** Expression of proinflammatory cytokines (TNF-α, IL-6) in RAW264.7 cells transfected with lentivirus oe-SNHG1 treated with LPS, as determined by ELISA. **(M)** Bacterial killing assay was performed in RAW264.7 cells transfected with lentivirus sh-SNHG1 treated with LPS or not. Data are indicated as the mean ± SD, *P<0.05, **P<0.01, ***P<0.001, ****P<0.0001.

To determine the functional role of SNHG1 in LPS-mediated inflammatory and immune responses in macrophages, SNHG1 expression was silenced and overexpressed by lentivirus-mediated gene modification. After LPS stimulation, the levels of proinflammatory cytokines (TNF-α, iNOS, IL-18, MCP-1, IL-6 and IL-1β) were significantly increased (**Figures 3A, B**). Protein expression appeared later than gene expression, so we stimulated RAW264.7 cells with LPS at least 24 h, and the concentrations of TNF-α and IL-6 were found to be significantly increased (**Figures 3C, D**). To determine the effects of SNHG1 on macrophage polarization, stably transfected RAW264.7 cells infected with LV-NC or LV-SNHG1 were constructed, and SNHG1 was significantly reduced (**Figure 3E**). The levels of proinflammatory cytokines (TNF-α, iNOS, IL-18, MCP-1, IL-6 and IL-1β) were significantly declined (**Figure 3F**). The protein levels of TNF-α and IL-6 were also significantly decreased at least 24 h when SNHG1 expression was silenced (**Figures 3G, H**). Similarly, SNHG1-overexpressing RAW264.7 cells were constructed (**Figure 3I**), and the levels of proinflammatory cytokines (TNF-α, iNOS, IL-18, MCP-1, IL-6 and IL-1β) were significantly increased (**Figure 3J**). The protein levels of TNF-α and IL-6 were significantly increased at 24 h (**Figures 3K, L**). Consistent with the diminished production of these cytokines, LPS-treated macrophages with SNHG1 knockdown also demonstrated reduced bactericidal activity (**Figure 3M**), a characteristic of M1 activation.

### SNHG1 Interacts With HMGB1 in RAW264.7 Cells

**Figure 4:**
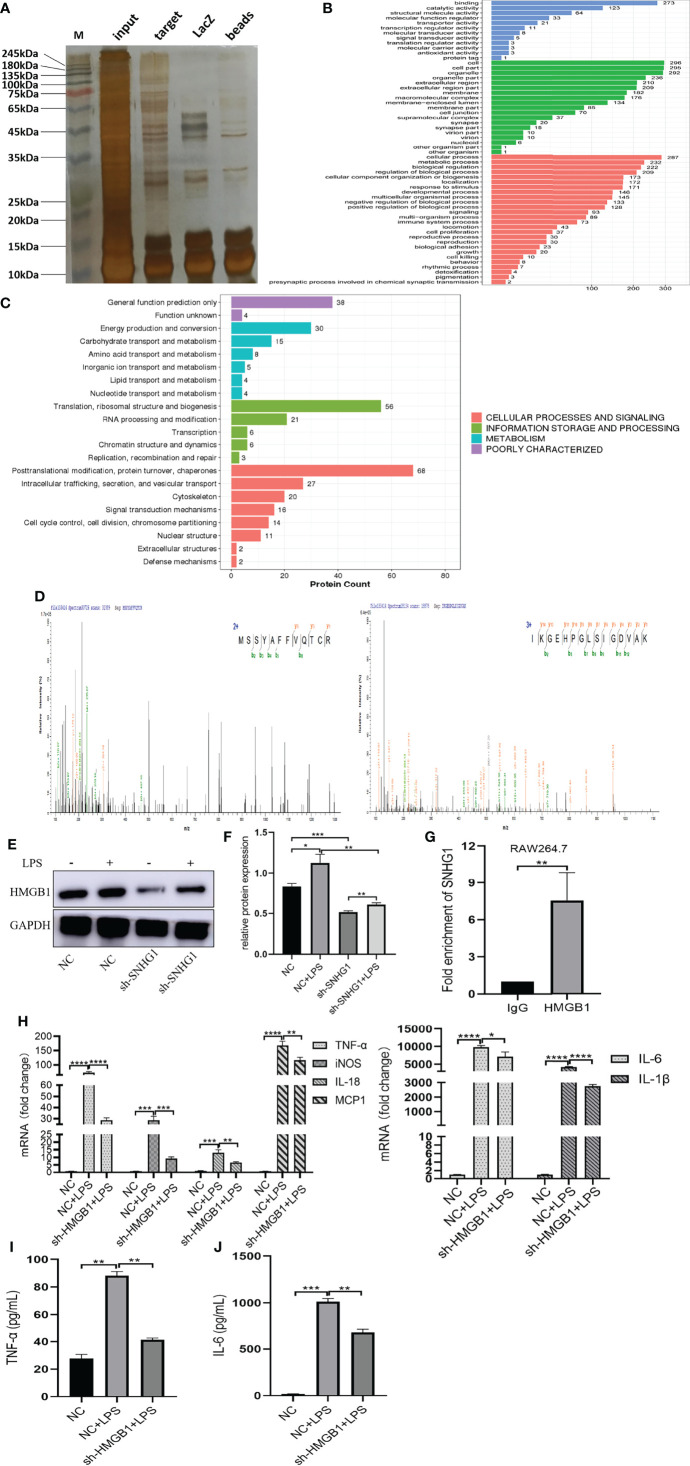
SNHG1 interacts with HMGB1 in RAW264.7 cells. **(A)** The results of the silver staining assay showed the SNHG1 ^binding^ protein. **(B)** GO function analysis of differentially expressed proteins that bind SNHG1. **(C)** KEGG pathway analysis of differentially expressed proteins that bind SNHG1. **(D)** Two HMGB1 peptides were detected in the mass spectrometry results. **(E, F)** The expression of HMGB1 in RAW264.7 cells treated with LPS, as determined by Western blot. **(G)** Anti-HMGB1 RIP assay was executed in RAW264.7 cells, followed by qRT-PCR. **(H)** Expression of proinflammatory cytokines (TNF-α, iNOS, IL-18, MCP-1, IL-6 and IL-1β) in RAW264.7 cells transfected with lentivirus sh-HMGB1 treated with LPS, as determined by qRT-PCR. **(I, J)** Expression of proinflammatory cytokines (TNF-α, IL-6) in RAW264.7 cells transfected with lentivirus sh-HMGB1 treated with LPS, as determined by ELISA. Data are indicated as the mean ± SD, *P<0.05, **P<0.01, ***P<0.001, ****P<0.0001.

To explore the molecular mechanism underlying the proinflammatory activity of SNHG1 in LPS-induced macrophage activation, we performed ChIRP assays to identify the proteins associated with SNHG1 in LPS-induced RAW264.7 cells. As shown in **Supplementary Figure 1**, the results verified the RNA probe enrichment effect. The results of the silver staining assay showed the SNHG1 binding protein (**Figure 4A**). The potential interacting proteins are shown in **Supplementary Data 1**. To clarify the biological functions of putative proteins on SNHG1, GO and KEGG pathway analyses of the involved targets were conducted (**Figures 4B, C**) after confirmation *via* experiments. We observed that SNHG1 was specifically associated with HMGB1. The results from SNHG1 ChIRP assays showed specific bands at ~25 KD *via* mass spectrometry. As shown in **Figure 4D**, two HMGB1 peptides were detected in the mass spectrometry results. Silencing SNHG1 expression significantly reduced the expression of HMGB1 (**Figures 4E, F**). Moreover, RIP assays showed that the antibodies of HMGB1 could significantly enrich SNHG1 (**Figure 4G**), whereas the IgG control could not. Then, the effect of HMGB1 on M1 polarization of macrophage was further evaluated using qRT-PCR and ELISA. As shown in **Supplementary Figures 1A**–**C**, the LV3-HMGB1-551 was efficiently reduced the expression of HMGB1 in RAW264.7. The levels of proinflammatory cytokines (TNF-α, iNOS, IL-18, MCP-1, IL-6 and IL-1β) were significantly declined when HMGB1 expression was silenced (**Figure 4H**). The protein levels of TNF-α and IL-6 were also significantly decreased when HMGB1 expression was silenced (**Figures 4I, J**). Together, these results indicate that SNHG1 specifically binds with HMGB1 in LPS-induced RAW264.7 cells and promote the classical activation of macrophages and inflammatory reaction.

### The Function of SNHG1 as a Downstream Mediator

**Figure 5:**
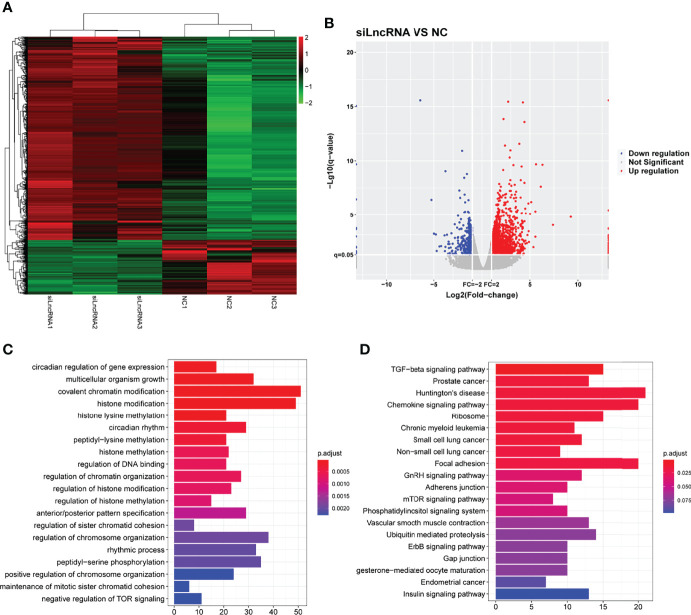
The functional of SNHG1 in downstream mediator. **(A)** The expression of downstream mRNAs in RAW264.7 cells transfected with lentivirus sh-SNHG1 in a heatmap. **(B)** The expression of upregulated and downregulated mRNAs in a volcano plot. **(C)** GO function analysis of differentially expressed mRNAs. **(D)** KEGG pathway analysis of differentially expressed mRNAs.

To verify the effect of SNHG1 on downstream mediators, transcriptome profiling was performed using RNA sequencing in RAW264.7 cells transfected with si-SNHG1 to investigate the related signaling pathways in RAW264.7 cells. Then, in comparison with the NC group, the depletion of SNHG1 affected the expression levels of 584 genes, which was shown by a hierarchical clustering heatmap (fold-change >2, **Figure 5A** and **Supplementary Data 2**). Among all the differentially expressed genes (DEGs), 518 genes were upregulated and 66 genes were downregulated (fold-change >2, **Figure 5B**). Finally, we performed GO and KEGG pathway analyses to highlight the up- and downregulation of the two groups of genes. As shown in **Figures 5C, D**, GO functional annotation and KEGG enrichment showed that differentially expressed genes were mainly involved in protein modification and chemokine signaling pathways.

### Screening and Biological Function Analysis of DEGs in ALI Mice

**Figure 6:**
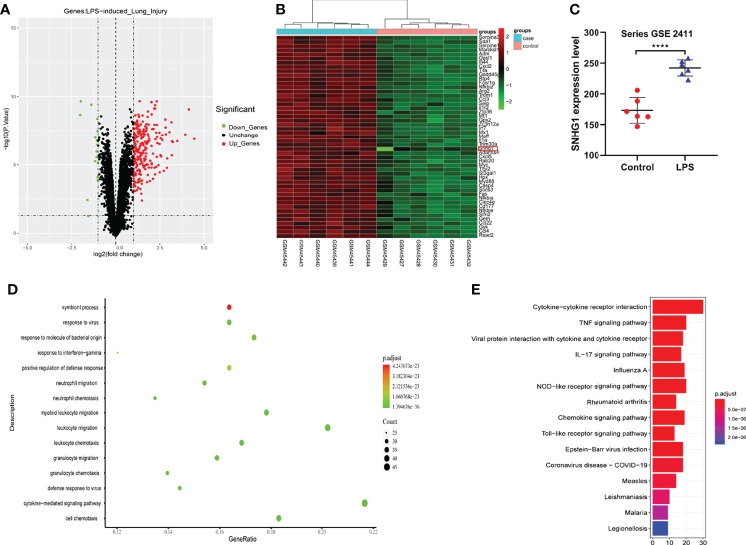
Screening and biological function analysis of DEGs in ALI mice in the GEO database. **(A)** The expression of upregulated and downregulated genes in ALI mice in a volcano plot. **(B)** The expression of SNHG1 was upregulated among the upregulated genes. **(C)** Comparison with the control group. SNHG1 expression in the ALI mouse model. **(D)** GO function analysis of upregulated genes in the ALI mouse model. **(E)** KEGG pathway analysis of upregulated genes in the ALI mouse model. Data are indicated as the mean ± SD, ****P<0.0001.

Given that SNHG1 was involved in the inflammatory response and macrophage activation, screening and biological function analysis of DEGs in ALI mice was performed. After GSE2411 (GEO database) screening, 249 DEGs, including 233 upregulated genes and 16 downregulated genes, were identified, as shown in **Figure 6A** and **Supplementary Data 3** and **4**. Among the upregulated genes, we found that SNHG1 expression was upregulated (**Figure 6B**). In comparison with the control group, SNHG1 expression was significantly increased (**Figure 6C**). As shown in **Figure 6D**, the main enriched GO terms of LPS vs. control in upregulated genes included response to virus, response to molecule of bacterial origin, neutrophil migration, neutrophil chemotaxis and so on. Analogously, KEGG enrichment analysis showed that the main enriched pathways were associated with inflammation, including cytokine−cytokine receptor interaction, TNF signaling pathway, IL−17 signaling pathway, chemokine signaling pathway and so on (**Figure 6E**). Together, the analysis showed that these important differentially expressed genes were involved in the immune and inflammatory responses.

### Tanreqing Injection (TRQ) Inhibits Inflammatory Responses in LPS-Activated Macrophages

**Figure 7:**
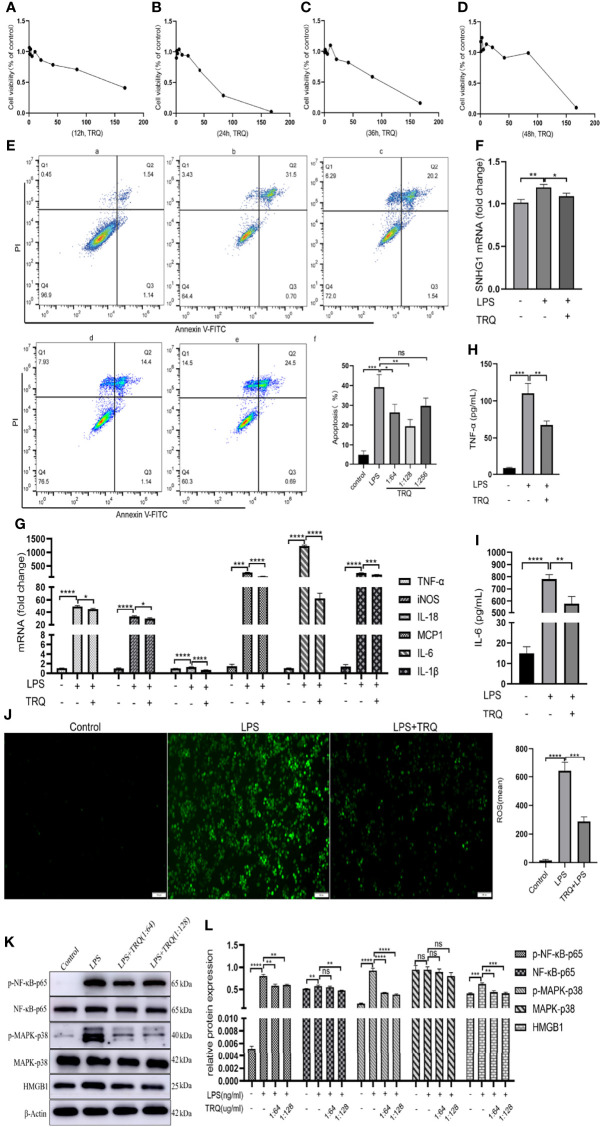
Tanreqing injection (TRQ) inhibits inflammatory responses in LPS-activated macrophages. **(A–D)** The toxicity of TRQ at different concentrations in RAW264.7 cells, as determined by CCK-8 assay. **(E)** Apoptosis assays of RAW264.7 cells treated with TRQ at concentrations of 1:64, 1:128 and 1:256. **(F)** The expression of SNHG1 in LPS-activated macrophages after TRQ treatment, as determined by qRT-PCR. **(G)** Expression of proinflammatory cytokines (TNF-α, iNOS, IL-18, MCP-1, IL-6 and IL-1β) in LPS-activated RAW264.7 cells after TRQ treatment, as determined by qRT-PCR. **(H, I)** Expression of proinflammatory cytokines (TNF-α, IL-6) in LPS-activated RAW264.7 cells after TRQ treatment, as determined by ELISA. **(J)** Production of ROS in RAW264.7 cells after LPS and TRQ treatment (magnification, ×100, scale bar, 100 μm). **(K, L)** Relative protein levels of p-NF-κB-p65, NF-κB-p65, p-MAPK-p38, MAPK-p38 and HMGB1 were detected in LPS-induced RAW264.7 cells treated with TRQ. Data are indicated as the mean ± SD, ns *P*≥0.05, *P<0.05, **P<0.01, ***P<0.001, ****P<0.0001.

Previous research has shown that TRQ can regulate immunity and inhibit oxidative stress, inflammation and cell apoptosis in ALI (25). To verify that TRQ inhibits LPS-induced inflammatory responses, we used LPS-stimulated macrophages. First, we determined the toxicity of TRQ at different concentrations. TRQ was diluted by using DMEM to a series to concentrations including 1:2, 1:4, 1:8, 1:16, 1:32, 1:64, 1:128, 1:256, 1:512 by volume on RAW264.7 cells, and then stimulate those cells at least 12 h, 24 h, 36 h and 48 h (**Figures 7A**–**D**). According to the concentrations, we selected 1:64, 1:128 and 1:256 to carry out cell apoptosis. As shown in **Figure 7E**, apoptosis assays revealed that TRQ markedly decreased the percentages of apoptotic cells relative to the control group, and a concentration of 1:128 had a particularly obvious effect. Then, we chose a concentration of 1:128 for the following experiment. TRQ effectively reduced the expression of SNHG1 in LPS-activated macrophages (**Figure 7F**). The levels of proinflammatory cytokines (TNF-α, iNOS, IL-18 MCP-1, IL-6 and IL-1β) (**Figure 7G**) and the protein levels of TNF-α and IL-6 were significantly reduced by TRQ (**Figures 7H, I**). TRQ markedly decreased the production of ROS in RAW264.7 cells relative to LPS group (**Figure 7J**). We also found that the expression of p-NF-κB-p65, p-MARK-P38 and HMGB1 was significantly reduced by TRQ (**Figures 7K, L**). Taken together, these results show that TRQ can alleviate the inflammatory response through the NF-kB/SNHG1/HMGB1 axis.

### TRQ Mitigates LPS-Induced ALI

**Figure 8:**
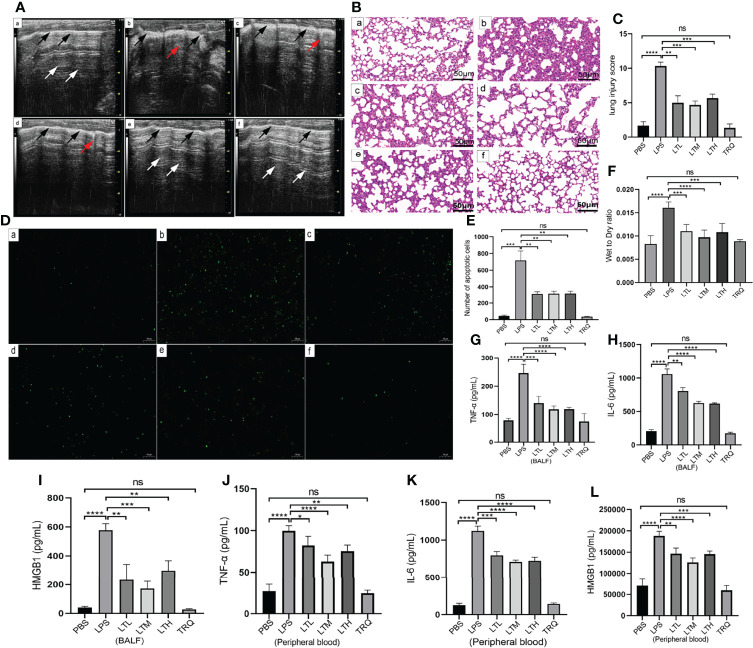
TRQ mitigates LPS-induced ALI. **(A)** Ultrasound imaging was performed to assess lung injury in the control **(a)**, LPS **(b)**, LTL **(c)**, LTM(d), LTH **(e)** and TRQ **(f)** groups. **(B)** HE staining was performed to assess lung injury in the control **(a)**, LPS **(b)**, LTL **(c)**, LTM **(d)**, LTH **(e)** and TRQ **(f)** groups (magnification, ×200, scale bar, 50 μm). **(C)** The lung injury score of HE staining. **(D)** The TUNEL apoptosis assay of lung tissue in the control **(a)**, LPS **(b)**, LTL **(c)**, LTM **(d)**, LTH **(e)** and TRQ **(f)** groups (magnification, ×100, scale bar, 100 μm). **(E)** The apoptosis cells number of lung tissue. **(F)** The wet/dry weight ratio of right upper lung tissue in the control, LPS, LTL, LTM, LTH and TRQ groups. **(G–I)** Expression of proinflammatory cytokines (TNF-α, IL-6 and HMGB1) in the BALF, as determined by ELISA. **(J–L)** Expression of proinflammatory cytokines (TNF-α, IL-6 and HMGB1) in the peripheral blood supernatant, as determined by ELISA. Data are indicated as the mean ± SD, ns, *P* ≥0.05, *P<0.05, **P<0.01, ***P<0.001, ****P<0.0001.

To determine whether TRQ could protect against LPS-induced ALI, ultrasound imaging and HE staining were performed to assess lung injury and the pathological changes in the lung after 24 h of LPS treatment. Ultrasound characteristics of normal lung tissue are lung sliding with parallel horizontal lines below the pleural line, called A-lines (26). B lines are defined as comet tail-like hyperechoic reverberation artifacts arising from and perpendicular to the pleural line, which is representative of thickened interlobular septa. As shown in **Figure 8A**, lung tissues in healthy mice showed A lines (white arrow) and a uniformly continuous pleural line (black arrow), whereas multiple well-defined B lines and thickened pleural and ground-glass areas were observed in the LPS-induced mouse model. More B lines and ground-glass areas were observed in the LPS group than in the PBS group, while fewer B lines and ground-glass areas were observed in the LPS+4 ml/kg TRQ, LPS+6 ml/kg TRQ and LPS+8 ml/kg TRQ groups than in the LPS treatment group. These data reasonably suggest that TRQ mitigates alveolar-interstitial edema and thickened interlobular septa. Similarly, HE staining was performed to assess the pathological changes in the lung. Compared to the control group, more serious alveolar edema, pulmonary hemorrhage, atelectasis and inflammatory cell infiltration were observed in the LPS-induced mouse model, and notably, these phenomena were markedly alleviated after TRQ treatment (**Figure 8B**). As **Figure 8C** shows, the lung injury score was consistent with the pathological section. Next, we further explored how TRQ plays a role in deterring the development of ARDS induced by LPS. Cell apoptosis was applied to assess the severity. In accordance with the above data, TRQ also hampered cell apoptosis (**Figure 8D**), and the number of apoptotic cells is shown (**Figure 8E**). LPS also increased the lung wet/dry weight ratio compared with that of the control group and was mitigated by TRQ (**Figure 8F**). Furthermore, the expression levels of the proinflammatory cytokines TNF-α, IL-6 and HMGB1 in the BALF and peripheral blood supernatant were measured by ELISA. Compared to the control group, the mice treated with LPS had significantly increased levels of the cytokines TNF-α, IL-6 and HMGB1, and these proinflammatory cytokines were alleviated by TRQ (**Figures 8G**–**L**). These results illustrated that administration of TRQ could alleviate LPS-induced lung injury in mice.

### TRQ Regulates the Activation of M1 Macrophages and the Expression of SNHG1

**Figure 9:**
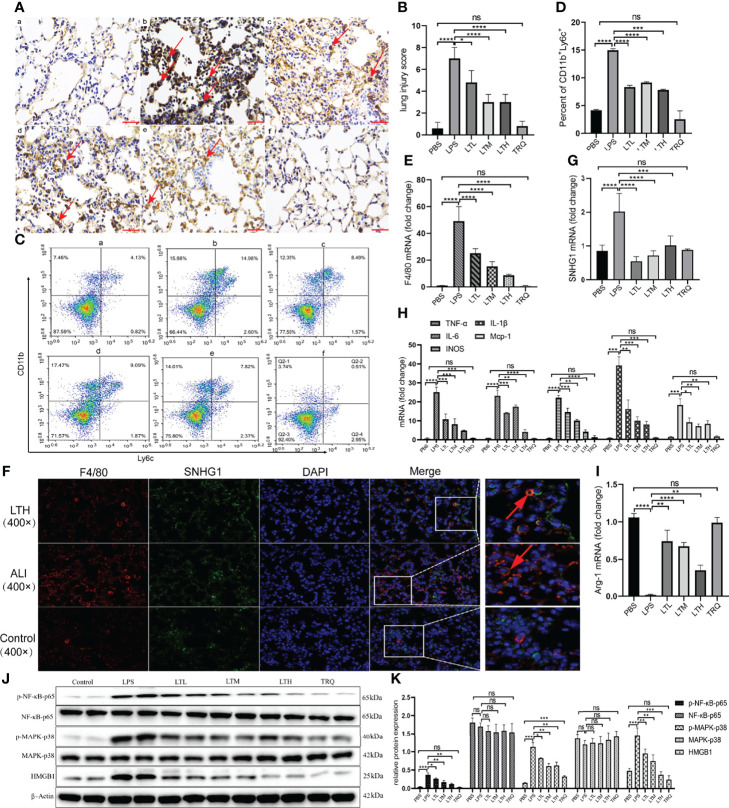
TRQ regulates the activation of M1 macrophages and the expression of SNHG1. **(A, B)** IHC of F4/80 was performed to show the expression of macrophages in the control **(a)**, LPS **(b)**, LTL **(c)**, LTM **(d)**, LTH **(e)** and TRQ **(f)** groups (magnification, ×100, scale bar, 100 μm). **(C, D)** The percentage of F4/80+Ly6c+ M1 macrophages in the control **(a)**, LPS **(b)**, LTL **(c)**, LTM **(d)**, LTH **(e)** and TRQ **(f)** groups, as determined by flow cytometry. **(E)** The expression of F4/80 in the control, LPS, LTL, LTM, LTH and TRQ groups, as determined by qRT-PCR. **(F)** The expression of SNHG1 (green) was expressed on F4/80 (red) macrophages in control, ALI, LTH group, as determined by FISH assay. **(G)** The expression of SNHG1 in the control, LPS, LTL, LTM, LTH and TRQ groups, as determined by qRT-PCR. **(H)** The expression of proinflammatory cytokines (TNF-α, iNOS, IL-18, MCP-1, IL-6 and IL-1β) in the control, LPS, LTL, LTM, LTH and TRQ groups, as determined by qRT-PCR. **(I)** Anti-inflammatory cytokines (Arg-1) in the control, LPS, LTL, LTM, LTH and TRQ groups, as determined by qRT-PCR. **(J, K)** The expression of NF-κB-p65, p-NF-κB-p65, MARK-P38, p-MARK-P38 and HMGB1 in the in the control, LPS, LTL, LTM, LTH and TRQ groups, as determined by Western Blot. Data are indicated as the mean ± SD, ns, *P* ≥0.05, *P<0.05, **P<0.01, ***P<0.001, ****P<0.0001.

Previous data provide intuitive evidence that TRQ exerts protective effects against ALI. We further studied the specific mechanism by which TRQ reduces acute lung injury. After the administration of intratracheal LPS, IHC of F4/80 showed upregulation of macrophages in the lung and reduction by TRQ (**Figures 9A, B**). We also investigated the ratio of monocytes in the peripheral blood by flow cytometry. After LPS injection, the percentage of F4/80^+^Ly6c^+^ M1 macrophages was significantly increased, and the ratio significantly reduced after TRQ administration (**Figures 9C, D**). The expression of F4/80 was significantly increased in the ALI group and reduced after TRQ administration (**Figure 9E**). FISH assay confirmed that SNHG1 (green) was expressed on F4/80 (red) macrophages and mainly collocated in cytoplasm in control, ALI, LTH group (**Figure 9F**). qRT-PCR of SNHG1 showed a significant reduction after TRQ administration compared with the ALI group (**Figure 9G**). The proinflammatory M1 marker genes (TNF-α, IL-6, IL-1β, iNOS and MCP-1) were significantly increased (**Figure 9H**), whereas the anti-inflammatory M2 marker gene (Arg-1) was significantly reduced in the lungs of ALI mice (**Figure 9I**). Conversely, TRQ (4 ml/kg, 6 ml/kg and 8 ml/kg) administration inhibited LPS-induced M1 marker gene expression (TNF-α, IL-6, IL-1β, iNOS and MCP-1) and enhanced M2 marker gene expression (Arg-1) in the lungs of ALI mice (**Figures 9H, I**). We also found that the expression of p-NF-κB-p65, p-MARK-P38 and HMGB1 was significantly increased in the ALI group and reduced after TRQ administration (**Figures 9J, K**). These results illustrated that administration of TRQ could regulate the activation of M1 macrophages and the expression of SNHG1 in an ALI mouse model.

## Discussion

In the present study, we showed that TRQ alleviates LPS-induced ALI. TRQ decreased the infiltration and activation of macrophages and mitigated inflammation of the lungs in ALI mice. Further mechanistic studies showed that TRQ suppressed SNHG1 at least partially through the NF-kB signaling pathway. Collectively, these data suggest that TRQ may be valuable for the treatment of ARDS and that SNHG1 may be a potential diagnostic marker of the severity of illness in ARDS.

The regulation of macrophage activation has been under extensive investigation, particularly at the transcriptional and epigenetic levels (8, 27, 28). The latest research has shown that miRNAs are involved in a variety of pathogeneses by regulating macrophage activation (29). However, although a much larger number of lncRNAs is found in mammalian cells, the role of regulating macrophage activation remains poorly understood. Recent studies have shown that SNHG1 is dysregulated in many complicated inflammatory diseases. For instance, a study showed that SNHG1 was remarkably elevated in LPS-induced BV2 cells, and its depletion mitigated neuroinflammation through the miR-7/NLRP3 axis in Parkinson’s disease (16). Similarly, research has shown that SNHG1 is significantly upregulated in LPS-induced PC12 cells and that the depletion of SNHG1 can mitigate LPS-induced cell apoptosis and autophagy (30). To clarify the role of SNHG1 in macrophage activation, we initially determined the regulation of its expression by LPS and IL-4, which are classically used to induce the two polarized states of macrophages. SNHG1 expression was significantly upregulated in LPS-induced RAW264.7 macrophages and downregulated in IL-4-induced RAW264.7 macrophages. We also demonstrate that SNHG1 is a key player in controlling macrophage phenotype. SNHG1 not only promotes M1 macrophage activation but also inhibits M2 macrophage formation. Depletion of SNHG1 could reduce the expression of proinflammatory cytokines such TNF-α, IL-6, and iNOS.

Activation of the LPS-NF-kB pathways transcriptionally promoted SNHG1 expression, which was mainly enriched in the cytoplasm. The NF-κB family of transcription factors has an essential role in inflammation and innate immunity. Previously, it was reported that miRNA-21, miR-155 and lncRNA-Malat1 were upregulated by NF-κB and promoted inflammation (17, 31, 32). In this study, we found that NF-κB subunit p65 bound to the SNHG1 promoter in LPS-treated macrophages and indicated that SNHG1 was a direct transcriptional target of LPS-induced NF-κB activation. NF-κB can directly increase the expression of SNHG1. We also found that the expression of p-NF-κB-p65 and p-MARK-P38 was significantly increased in the ALI group and reduced after TRQ administration.

LncRNAs typically exert their biological functions through physical interactions with regulatory proteins, miRNAs or other cellular factors. For instance, a study showed that LINC01138 physically interacts with PRMT5 and exerts its oncogenic activity by stabilizing PRMT5 in HCC cells (33). The long noncoding RNA MEG3 is able to sponge microRNA-7b to upregulate the expression of NLRP3 to promote the aggravation of acute lung injury (34). lincRNA-Cox2 can also bind NF-κB p65 and promote its nuclear translocation and transcription, modulating the expression of the inflammasome sensor NLRP3 and adaptor ASC (35). In this study, we identified that SNHG1 promoted the inflammatory responses induced by LPS through combining with HMGB1 and demonstrated the proinflammatory functions of the NF-kB-SNHG1-HMGB1 axis in LPS-induced RAW264.7 macrophage cells. Notably, we identified HMGB1 as the downstream effector of SNHG1 and promoted the inflammatory response. As a proinflammatory cytokine, HMGB1 is a ubiquitous nuclear and cytosolic protein and is released into the circulation (36). Necrotic cells release HGMB1, but HMGB1 is also actively secreted by inflammatory macrophages. HMGB1 plays an important role in activating inflammatory responses directly through PRRs, including TLR2 and TLR4, and advanced glycation end-product receptor (AGER) (37). HMGB1 promotes macrophage polarization and negatively influences phagocytosis of apoptotic cells and activates inflammatory responses (38). In our study, we also found that HMGB1 knockdown inhibited LPS-induced the classical activation of macrophages and inflammatory reaction. Previous research found that HMGB1 plays a critical role in inducing sepsis in humanized mice, most likely by inducing a human cytokine storm, and silencing HMGB1 after the development of symptoms can reverse sepsis in humanized mice (39). A large body of evidence indicates that HMGB1 is also required for the development or progression of inflammation, even in the absence of infection, such as autoimmune arthritis, sterile hepatic necrosis, and other conditions that lead to inflammation and tissue injury (40). Billiar and colleagues discovered that HMGB1 is released during sterile ischemia-reperfusion injury and, as a proximal trigger, induces the release of other classically associated cytokines mediating sickness responses, including TNF, IL-1, and IL-6 (41, 42). In our study, we also found that the expression of HMGB1 was significantly increased in LPS-induced RAW264.7 and the ALI group and reduced after TRQ administration.

Traditional Chinese medicine (TCM) formulas typically have bidirectional regulatory effects with multiple functional herbs that restore the whole body to a balanced state. The characteristic advantage of TCM treatment is to mobilize the patient’s self-adaptation and disease resistance and treat it based on syndrome differentiation (43). It can fight inflammation through multiple targets, multiple links, and multiple pathways and protect against lung injury. In recent years, clinical research and animal experiments of traditional Chinese medicine in the treatment of ALI/ARDS have continued to deepen, and certain results have been achieved. ALI/ARDS is inferred based on clinical symptoms and is equivalent to “febrile disease” and “violent asthma” in traditional Chinese medicine, in which phlegm-heat and stasis are the pathological factors for the development of ALI/ARDS. TRQ is mainly used for severe pulmonary inflammation in the heat toxin-occluded stage and to treat phlegm-heat syndrome. At present, research on the mechanism of TRQ is still limited. One study found that in RAW264.7 macrophages treated with LPS, Scutellaria baicalensis in the TRQ prescription inhibits inflammation by preventing the NF-κB-induced secretion of Cox2 and iNOS, thereby inhibiting inflammation (4). In cytology study, we found that TRQ can reduce the apoptosis in LPS-induced RAW264.7 macrophages. Proinflammatory cytokines and the expression of SNHG1 were suppressed by TRQ. Oxidative stress refers to a state in which the production of total reactive oxygen species (ROS) in the body exceeds the defense capacity of the antioxidant system and the lung is one of the most vulnerable target organs (44). Therefore, ROS plays an important role in the ALI. In our study, we also found that TRQ can reduce the production of ROS.

ALI/ARDS are acute diffuse inflammatory lung injuries characterized by intractable hypoxemia and noncardiogenic pulmonary edema. Lung ultrasound examination has the advantages of high sensitivity, bedside examination, no radiation, and real-time evaluation (45). It is widely used in the evaluation of pulmonary edema in the intensive care unit. In ALI mouse model, we found that TRQ alleviate the alveolar-interstitial syndrome as well as pleural thickening. Given the spatial heterogeneity of lung lesions in ARDS, both of normal and abnormal artifacts can be observed in same image, we conduct HE staining to assess the pathological changes in the lung. Our data shown that compared to the control group, more serious alveolar edema, pulmonary hemorrhage, atelectasis and inflammatory cell infiltration were observed in the LPS-induced mouse model, and notably, these phenomena were markedly alleviated after TRQ treatment. To further prove the therapeutic effect, we also investigate the influence of TRQ on apoptosis, lung W/D weight ratio, release of cytokines from BALF and peripheral blood in ALI mouse model. Macrophages, as the main cells that recognize pathogen-related molecular patterns, trigger innate immunity and host defense, play an important role in ALI. Macrophages show different functional phenotypes in different microenvironments, which can be polarized into classically activated M1 macrophages and alternatively activated M2 macrophages. M1 macrophages produce high amounts of proinflammatory cytokines and are critical to the eradication of bacterial, viral and fungal infections (8–10). IHC of F4/80 was performed to show the expression of macrophages in the control, LPS, LTL, LTM, LTH and TRQ groups. After the administration of intratracheal LPS, IHC and qRT-PCR of F4/80 showed upregulation of macrophages in the lung and reduction by TRQ. FISH assay shows that SNHG is expressed in F4/80 macrophages. SNHG1 expression was significantly upregulated in LPS-induced mouse model, and reduction by TRQ. In response to macrophage depletion, monocytes always are recruited to the lung, where the microenvironment shapes them into closely resemble tissue-resident alveolar macrophages (46–48). We also investigated the ratio of monocytes in the peripheral blood by flow cytometry. After LPS injection, the percentage of F4/80+Ly6c+ M1 macrophages was significantly increased, and the ratio significantly reduced after TRQ administration.

## Conclusion

In conclusion, our findings showed that lncRNA SNHG1 was overexpressed, mainly enriched in the cytoplasm, and regulated by NF-κB in LPS-induced RAW264.7 cells. SNHG1 acts as a proinflammatory gene and significantly promotes the production of proinflammatory cytokines *in vitro* and *in vivo*. SNHG1 physically interacts with HMGB1 and exerts its proinflammatory activity by stabilizing HMGB1 in LPS-induced RAW264.7 cells; specific HMGB1 inhibitors can disrupt the association of SNHG1 and HMGB1. TRQ can inhibit the progression of inflammation by inhibiting NF-κB activation and may be a promising therapeutic drug for ALI/ARDS. SNGHG1 may be a promising prognostic indicator and could provide insight into ALI/ARDS disease progression.

## Data Availability Statement

The original contributions presented in the study are publicly available. This data can be found here: https://www.ncbi.nlm.nih.gov/geo/query/acc.cgi?acc=GSE190206.

## Ethics Statement

The animal study was reviewed and approved by The Ethics Committee of the First Affiliated Hospital of Zhengzhou University.

## Author Contributions

CH conceived and designed the studies, performed experiments, analyzed and interpreted the data, and drafted the manuscript. LX conceived and designed the studies. JL and CB provided critical advice on the study. YT, YL, and LQ performed the mouse experiments. JG, SZ, MY, and XL analyzed the data. All authors contributed to the article and approved the submitted version.

## Funding

This work was supported by the National Natural Science Foundation of China (Number 82074212) and the Science and Technology Department of Henan Province (CN) (Number SB201901036).

## Conflict of Interest

The authors declare that the research was conducted in the absence of any commercial or financial relationships that could be construed as a potential conflict of interest.

## Publisher’s Note

All claims expressed in this article are solely those of the authors and do not necessarily represent those of their affiliated organizations, or those of the publisher, the editors and the reviewers. Any product that may be evaluated in this article, or claim that may be made by its manufacturer, is not guaranteed or endorsed by the publisher.

## Supplementary Material

Supplementary 1 Summary of the potential interacting proteins with SNHG1.

Supplementary 2 Summary of the effects of SNHG1 depletion on the expression levels of 584 genes.

Supplementary 3 The 233 upregulated genes in ALI mice in the GEO database (GSE2411).

Supplementary 4 The 16 upregulated genes in ALI mice in the GEO database (GSE2411).

Supplementary Figure 1 RNA probe enrichment effect of SNHG1 in ChIRP assays.

Supplementary Figure 2 Silencing HMGB1 in RAW264.7 cells. **(A)** Effect of sh-HMGB1 lentivirus infection. **(B, C)** The efficiency of HMGB1 in RAW264.7 cells transfected with lentivirus sh-HMGB1-551 and sh-HMGB1-773 was determined by qRT-PCR and Western Blot.
