# Supplementary figures and images for: Tanreqing Injection Attenuates Macrophage Activation and the Inflammatory Response via the lncRNA-SNHG1/HMGB1 Axis in Lipopolysaccharide-Induced Acute Lung Injury
Source: Front Immunol. 2022 Apr 25;13:820718. doi: 10.3389/fimmu.2022.820718 (PMC9084914; doi:10.3389/fimmu.2022.820718)

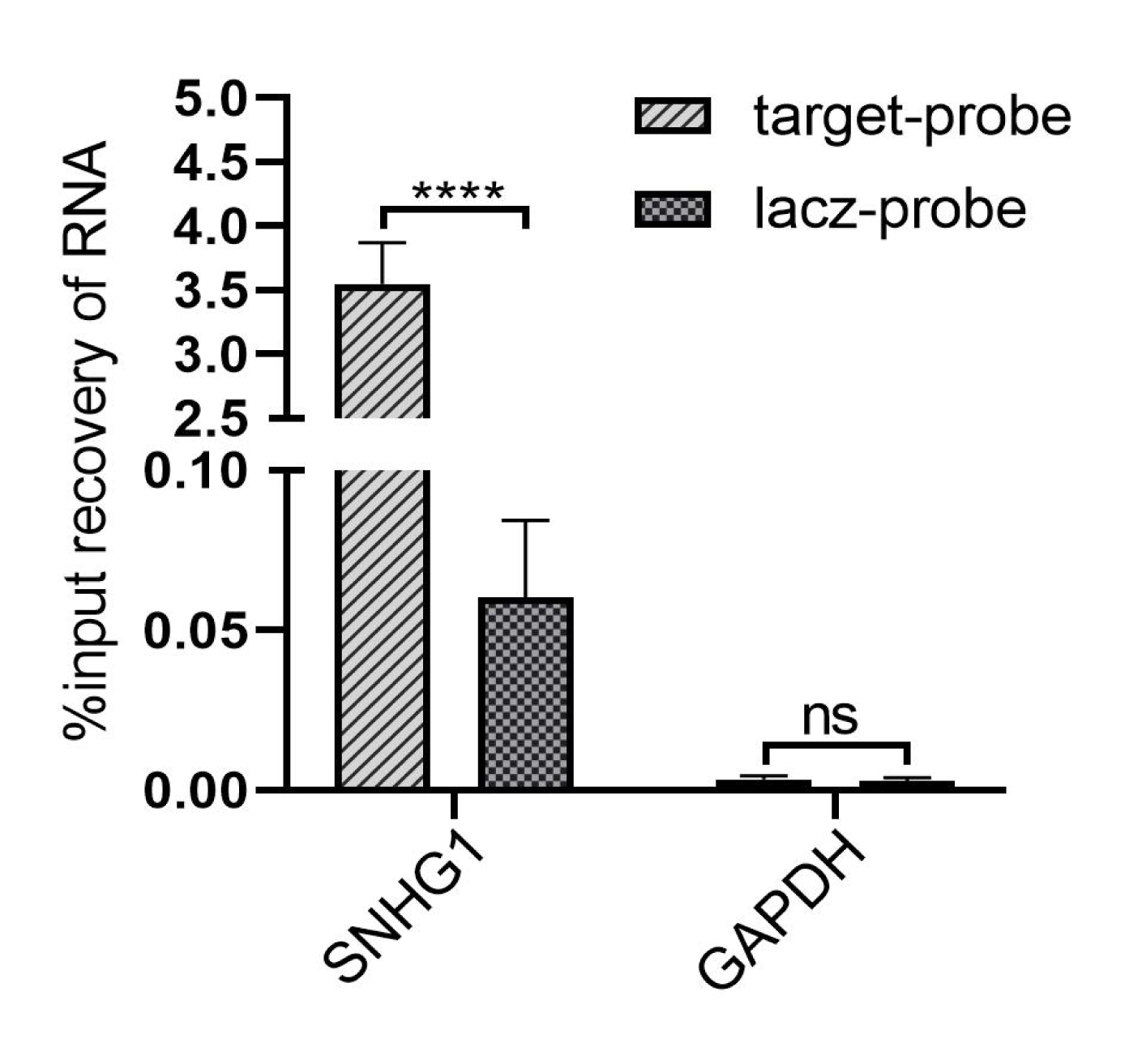

Supplement: Supplementary Figure 1 — RNA probe enrichment effect of SNHG1 in ChIRP assays. [file Image_1.tif]

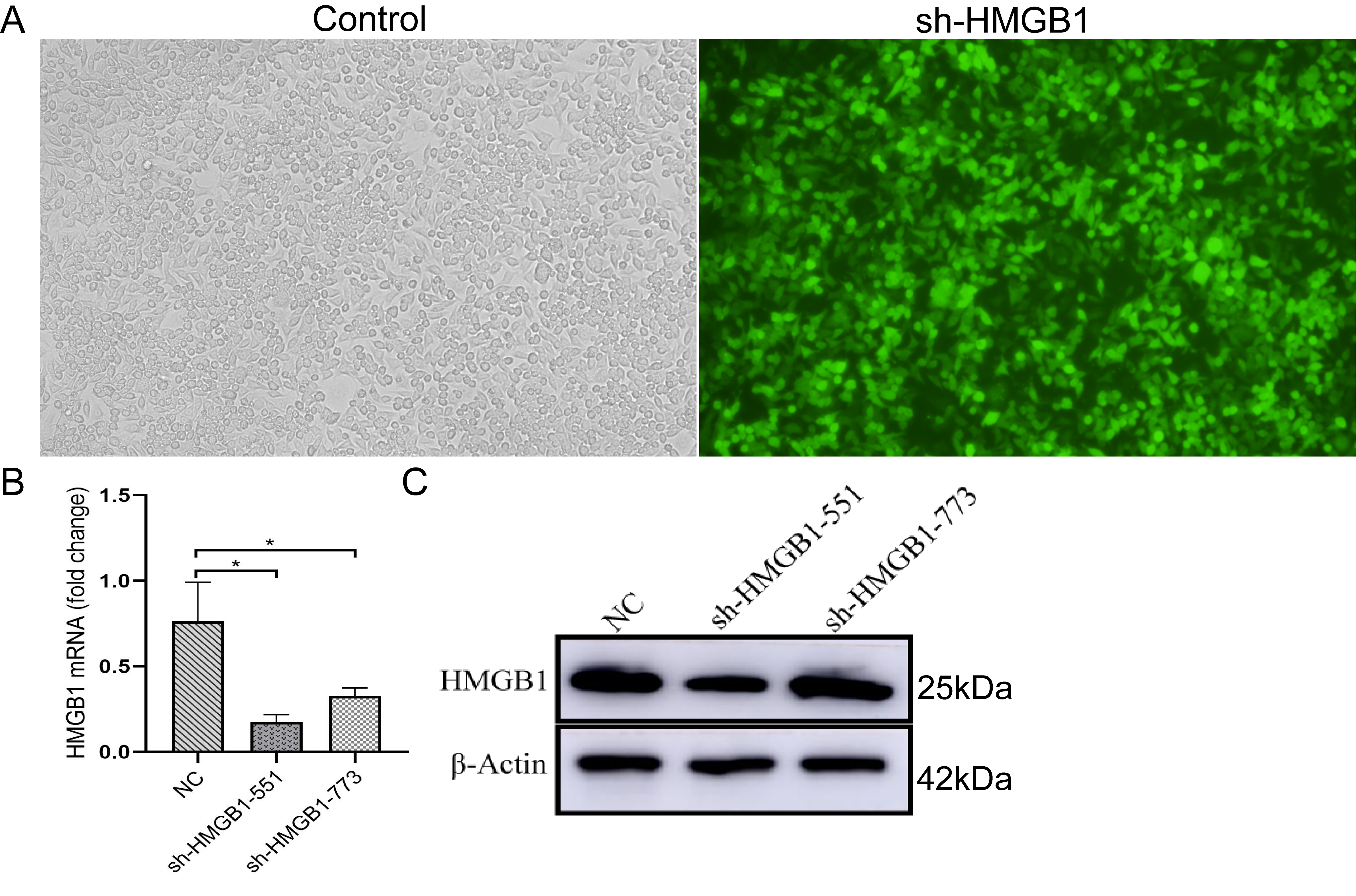

Supplement: Supplementary Figure 2 — Silencing HMGB1 in RAW264.7 cells. (A) Effect of sh-HMGB1 lentivirus infection. (B, C) The efficiency of HMGB1 in RAW264.7 cells transfected with lentivirus sh-HMGB1-551 and sh-HMGB1-773 was determined by qRT-PCR and Western Blot. [file Image_2.tif]
